# Characterisation and functionalisation of chitosan nanoparticles as carriers for double-stranded RNA (dsRNA) molecules towards sustainable crop protection

**DOI:** 10.1042/BSR20230817

**Published:** 2023-11-10

**Authors:** Dora Scarpin, Luca Nerva, Walter Chitarra, Loredana Moffa, Francesca D'Este, Marco Vuerich, Antonio Filippi, Enrico Braidot, Elisa Petrussa

**Affiliations:** 1 Department of Agriculture, Food, Environment and Animal Sciences (DI4A), University of Udine, Via delle Scienze 206, 33100 Udine, Italy; 2 Research Centre for Viticulture and Enology, Council for Agricultural Research and Economics (CREA-VE), Via XXVIII Aprile 26, 31015 Conegliano (TV), Italy; 3 Department of Medicine (DAME), University of Udine, P.le Kolbe 4, 33100 Udine, Italy

**Keywords:** Chitosan nanoparticles, dsRNAs, nanomaterial functionalization, nanoparticle/tegument interaction, RNA-interference, smart delivery

## Abstract

The need to minimise the impact of phytosanitary treatments for disease control boosted researchers to implement techniques with less environmental impact. The development of technologies using molecular mechanisms based on the modulation of metabolism by short dsRNA sequences appears promising. The intrinsic fragility of polynucleotides and the high cost of these techniques can be circumvented by nanocarriers that protect the bioactive molecule enabling high efficiency delivery to the leaf surface and extending its half-life.

In this work, a specific protocol was developed aiming to assess the best methodological conditions for the synthesis of low-size chitosan nanoparticles (NPs) to be loaded with nucleotides. In particular, NPs have been functionalised with partially purified Green Fluorescent Protein dsRNAs (*GFP* dsRNA) and their size, surface charge and nucleotide retention capacity were analysed. Final NPs were also stained with FITC and sprayed on *Nicotiana benthamiana* leaves to assess, by confocal microscopy, both a distribution protocol and the fate of NPs up to 6 days after application.

Finally, to confirm the ability of NPs to increase the efficacy of dsRNA interference, specific tests were performed: by means of *GFP* dsRNA-functionalised NPs, the nucleotide permanence during time was assessed both *in vitro* on detached wild-type *N. benthamiana* leaves and *in planta*; lastly, the inhibition of *Botrytis cinerea* on single leaves was also evaluated, using a specific fungal sequence (*Bc* dsRNA) as the NPs’ functionalising agent.

The encouraging results obtained are promising in the perspective of long-lasting application of innovative treatments based on gene silencing.

## Introduction

Within a very short time, the farming system is claimed to respond to several challenges in facing the predicted worldwide increase in food demand by 2050 [1]. A more sustainable agriculture, such as endorsed by the EU Commission for the Farm-to-Fork program, imposes a drastic reduction in chemical pesticides and fertilisers. Indeed, it calls for development and adoption of safer alternatives, respectful for the agro-ecosystems and the environment, with low-risk for human health [2].

During recent years, research and agriculture efforts have been largely devoted to improve the use of smart technologies and tools, such as naturally derived bio-stimulants, able to sustain high crop productivity with a low impact on the environment, improve plant nutrient use efficiency (NUE) [3,4] and abiotic stress tolerance [5] or boost the innate plant resistance to pathogens [6]. Among these bio-based compounds, bulk chitin and chitosan, its N-deacetylated derivative, have been gaining large interest. Due to the presence of their reactive amine and hydroxyl groups, they exert multiple bio-activities in a wide range of applications in crop protection, as well as in biotechnological and biomedical fields, cosmetics, and in food quality preservation [6–8].

Differently from chitin, chitosan application is not limited by a low solubility [9], since it is easily dissolved in water under acidic solution, while it represents a safe, non-toxic, bio-compatible and highly renewable bio-compound from fish production waste [10,11]. Bulk chitosan has been demonstrated to display several antimicrobial and antioxidant properties against pathogenic microorganisms when applied to different crops [6,12], as well as to act as bio-elicitor of natural plant immunity [13]. Furthermore, the reactive functional groups present in chitosan confer elevated biodegradability, high adsorption and gel-forming capacity [11,14], high facility in chemical cross-linking and chelation, entrapment and delivery of functional compounds and negatively charged biologically active macromolecules such as nucleotides and proteins [15]. As an alternative approach in integrated crop protection, innovative chitosan-based delivery systems and agro-nanochemicals have been recently developed [6,16,17]. Indeed, the encapsulation of pesticides and antimicrobial or active compounds in chitosan-formulated nanostructures offers several advantages, such as enhanced precise delivery into the target plant tissues, elevated bio-availability, stability and, in turn, a limitation in run-off of toxic compounds into the environment. Several chitosan nanoformulations, carrying conventional or systemic pesticides, bio-active plant-based extracts, DNA and Cu or Ag metals, have been used in recent years, demonstrating to be very effective systems for their uptake, translocation and delivery in a wide range of crop plants [17].

The very latest alternative to conventional agrochemicals in the research of chitosan-based nanotechnology is extended to the development of nanocarriers for genetically modified (GM)-free RNA interference (RNAi) technology. This is a conserved mechanism able to recognise endogenous as well as exogenous double-stranded RNA sequences (dsRNAs) and process them to smaller RNA molecules known as small interfering RNA (siRNAs) that lead to the degradation of homologous RNAs. Recent studies highlighted that RNAi is a natural protection strategy able to elicit plant defence responses against pathogens through post-transcriptional gene regulation [18–21]. Indeed, it has been already proven that induction of RNAi can be useful for pathogens control, such as viral, fungal, insect and nematode diseases [22–25]. Furthermore, the use of exogenously applied dsRNAs can be exploited to regulate plant endogenous genes to functionally characterise them or to impair fungal pathogens [26–28]. However, efficiency of exogenous naked dsRNAs applications can be limited by their low persistence in the environment (e.g. because of light, UV and thermal degradation) [29,30] and ability to overcome the physical barriers of the leaf surface [31]. Moreover, RNAs could be sequestered [32] or degraded within the plant cell [29].

In this regard, chitosan nanoparticle (NP) formulations, in addition to other efficient nano-assisted systems such as carbon structures, gold nanoclusters and DNA nanocarriers [33], could represent very promising tools for improving dsRNA delivery and stability, and overall response in RNAi-based crop protection strategies.

The study of the combination of these two strategies has recently been carried out by Xu and co-workers [34]. In this work, the effect of chitosan quaternary ammonium salt (HACC) nanoparticles complexed with dsRNAs targeting Tobacco Mosaic Virus (TMV) inhibitory sequences has been investigated. The HACC-dsRNA complex was characterised and its fate studied by means of fluorescent probes. Four different modes of delivery were tested *in planta*, observing that all of them were able to reduce the incidence of TMV infection.

In the present work, we trace some of the analyses carried out in the aforementioned research, concentrating, however: (a) on identifying the best method for NPs synthesis; (b) in defining the best formulation for foliar application also through NPs fluorescent labelling; (c) in the use of a single delivery mode (spray distribution), the most suitable for combating the target pathogen *Botrytis cinerea*. We firstly characterise different chitosan NP preparations by size and ζ-potential and we investigate their ability to be functionalised with nucleotide sequences as naked dsRNAs (*GFP* dsRNAs). Furthermore, the permanence of *GFP* dsRNA-NPs topically applied on wild-type *Nicotiana benthamiana* leaves is evaluated both in detached leaves under UV light treatment and in plants grown in controlled environment. To confirm the effectiveness also on pathogen defence, NPs conveying specific fungal sequences (*Bc* dsRNA) are subsequently employed for the growth inhibition of *B. cinerea* mycelium.

Finally, we perform confocal imaging of FITC-labelled chitosan nanostructures to track and visualise their adhesion and distribution on foliar surface of *N. benthamiana* and to assess their absorption up to 6 days after treatment.

## Results

In the present work, the application of the synthesis protocol of NPs by ionic gelation [35] aimed to improve the synthesis of functionalisable chitosan nanomaterials. Such products need to be as small as possible to meet the technical requirement of easy distribution on leaf surface, an adequate capacity of adhesion and high degree of stability. Moreover, in order to exploit the properties of chitosan, already known as a bio-agent, we verified the possibility to functionalise NPs with dsRNA polymers showing a phytoiatric effect.

A slight modification of the method suggested by Zhao and Wu [36] involves an oxidation treatment that partially degrades chitosan used to produce NPs, hereafter referred to as NPsD to distinguish them from NPsF obtained by the conventional method involving a preliminary chitosan filtration. The adjustment has led to a significant increase in the efficiency of synthesis if compared with original protocol, achieving a yield of 86.06 ± 1.50% instead of 62.58 ± 11.72% obtainable with the filtration process (*n*=6; *t*-test significance *P*=0.004)

**Figure 1:**
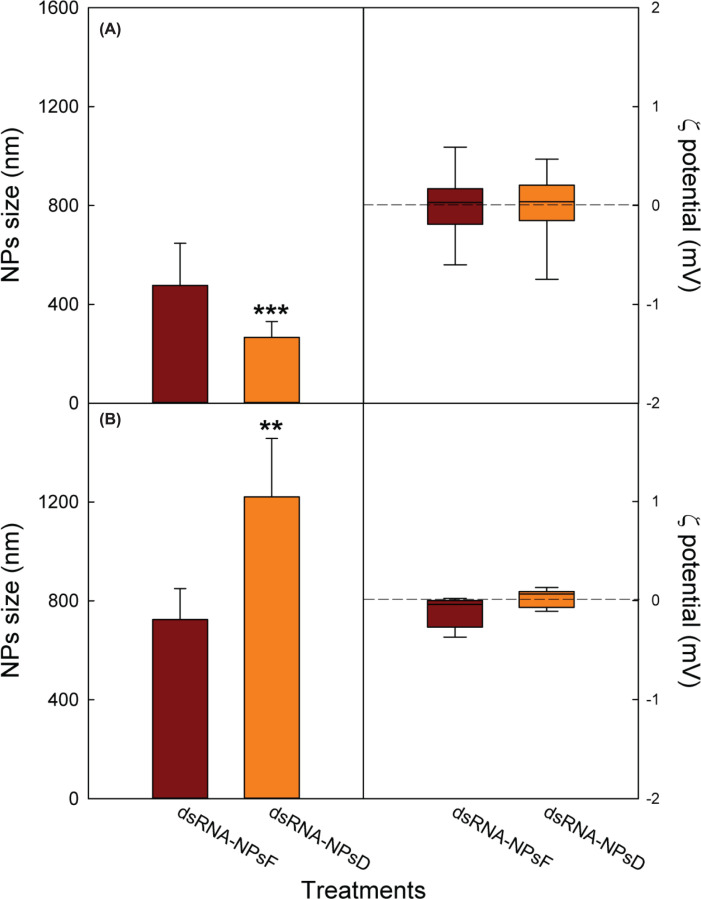
NPs characterisation Size and ζ potential of NPs obtained by the two preparations before (**A**) and after (**B**) their functionalisation with RNA. Data (*n*=6) are expressed: (left) as mean ± SD; (right) as boxplot whose whiskers correspond to the data range between minimum and maximum value, excluding outliers. The significance of the applied *T* test is ***P=0.000 (A) and **P=0.017 (B).

The characterisation by Dynamic Light Scattering (DLS) method showed a significant decrease in the size of NPsD in comparison with NPsF, whereas their ζ potential was substantially close to neutrality and unaffected by the synthesis treatment (Figure 1A). However, in both synthesis methods, the functionalisation with dsRNAs significantly led to an increase of the NP size compared with that of the respective non-functionalised NPs, where the degradation treatment provided a significant greater value than the filtration one (Figure 1B).

Again, the NPs functionalisation by dsRNA did not affect the surface charge, since it remained the same as measured in the empty NPs, regardless of the applied synthesis method.

The dsRNA retention efficiency of NPs obtained according to the two protocols was assessed by agarose gel electrophoresis and subsequent fluorescence analysis of the nucleotide-bound signal by means of UV lamp illumination.

**Figure 2:**
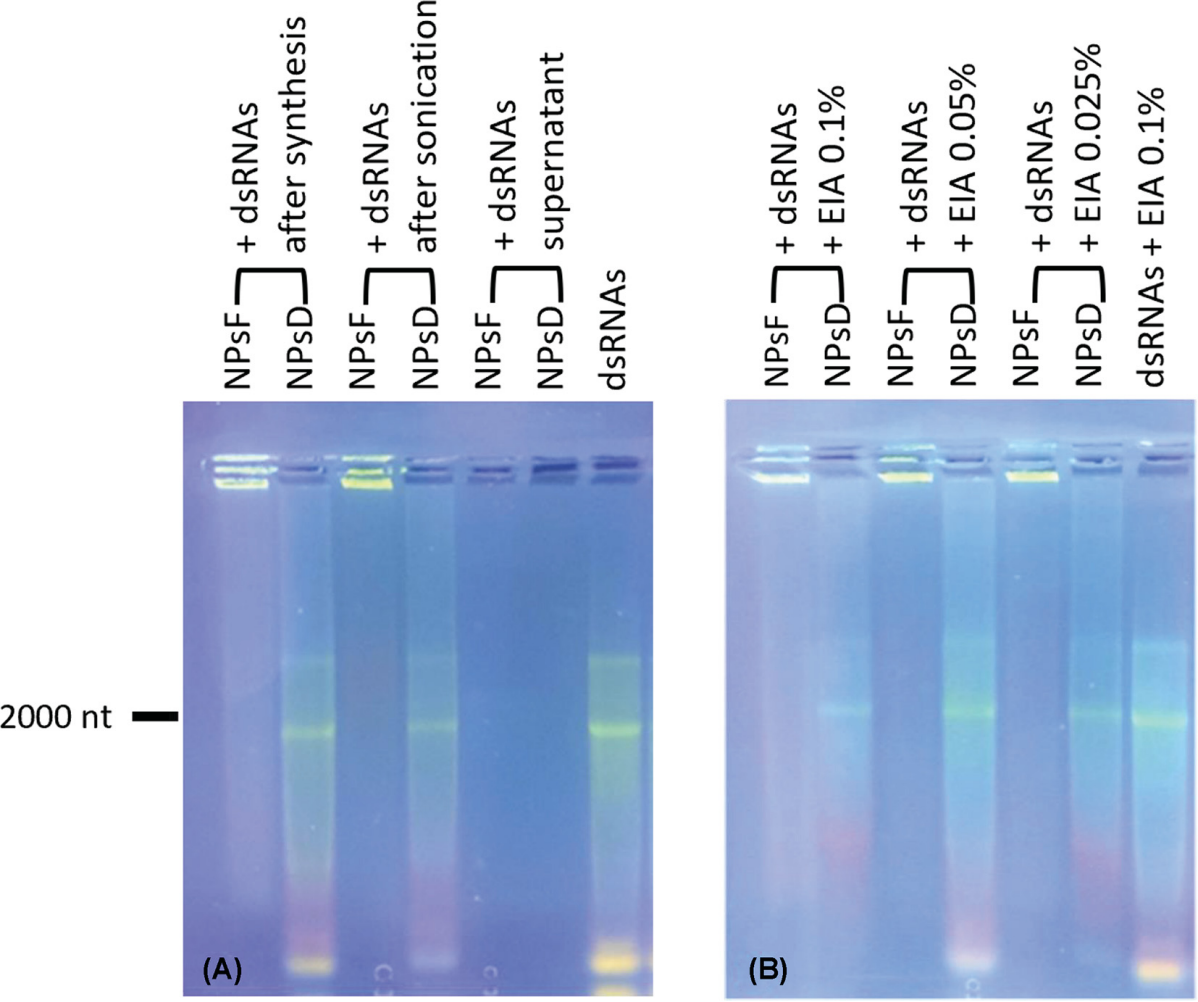
Gel electrophoresis on GFP dsRNA-loaded NPs Evaluation of the dsRNA retention capacity of NPs obtained by the two different preparations (**A**) and after their resuspension in different concentrations of EIA (**B**). The amount of free dsRNA was the same as that provided to functionalised NPs.

In Figure 2A, samples obtained by simple filtration of chitosan (NPsF) showed a much higher dsRNA retention capacity than those obtained by oxidative degradation (NPsD), if assayed either immediately after synthesis (lanes 1 and 2) or following refinement and sonication (lanes 3 and 4). This effect is confirmed by the high fluorescence signal of NPsF functionalised by dsRNAs observed at the well level (lanes 1 and 3), whereas in the case of NPsD samples (lanes 2 and 4) nucleotides migrated along the gel with a smear pattern, similar to that detectable in the free dsRNA sample (lane 7). In the lanes of the supernatants from the two NP preparations, no smear signal was detected, as a proof of the lack of dsRNA release from the NPs after synthesis.

A further electrophoretic run has been applied to test the effect of EIA (Figure 2B), a surfactant commonly used in agronomic practices, on the retention capacity of the NPs under study. After suspension in increasing concentrations of EIA, the NPsD show a release of the nucleotide polymers (lanes 2, 4 and 6) resembling that detectable in the free dsRNA sample resuspended in 0.1% EIA (lane 7) or in the samples without EIA (lanes 2 and 4, Panel A). In the loading wells of the latter, no fluorescent signal can be found anymore, indicating an almost complete runoff following electrophoresis. The release effect is maximised by the intermediate dose (0.05%) of surfactant, as evidenced by the increased fluorescence intensity of the electrophoretic bands. In contrast, NPsF samples show no release of nucleotides in the gel and the fluorescence signal is localised only at the loading point, in the starting well (Figure 2B, lanes 1, 3 and 5).

These results have led to identify the NPsF preparation as the best choice for the following tests involving NP application on *N. benthamiana* leaves.

According with the above-described results, the highest dose of EIA (0.1%) was chosen for the subsequent analyses on *N. benthamiana* leaves and plants. The further characterisation of the NPsF and the observation of their distribution pattern on the leaf were obtained by functionalisation with FITC probe and confocal microscopy analysis. For this purpose, FITC-doped NPsF have been characterised by DLS. As shown in Supplementary Figure S4, FITC-incorporation induced a significant decrease in the hydrodynamic diameter of NP size, as well as the addition of the wetting agent to the resuspension solution. On the contrary, neither FITC nor EIA influenced the surface charge of the NPs.

**Figure 3:**
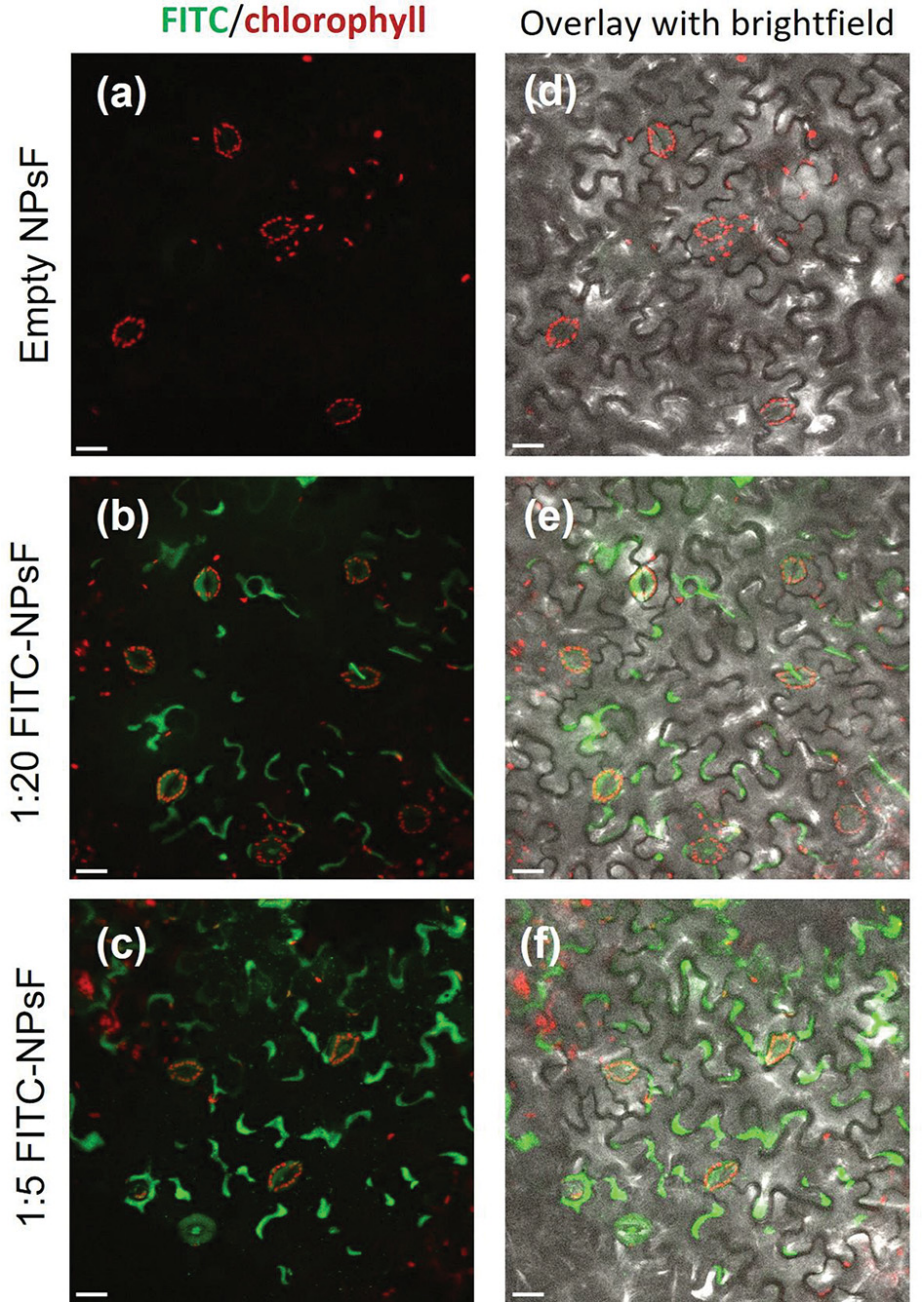
Confocal microscopy analysis of *N. benthamiana* abaxial side of leaf teguments sprayed with NPsF resuspended in 0.1% EIA Panels (**A,D**): Empty NPsF; panels (**B,E**): 1:20 FITC-NPsF; panels (**C,F**): 1:5 FITC-NPsF. Left column: maximum intensity projection micrographs (green channel, FITC; red channel, chlorophyll autofluorescence); right column: overlay with corresponding brightfield images; scale bar: 20 μm.

Confocal microscopy analysis highlighted the ability of NPsF to cover the leaf surface with a dose-dependent distribution according to the dilution level. The most (1:20) and moderately (1:5) diluted NPs were preferentially distributed along the tangential walls of the tegumental cells, as shown in Figure 3B–F. The presence of fluorescence signal was also detected within stomatal guard cells and hairs.

**Figure 4:**
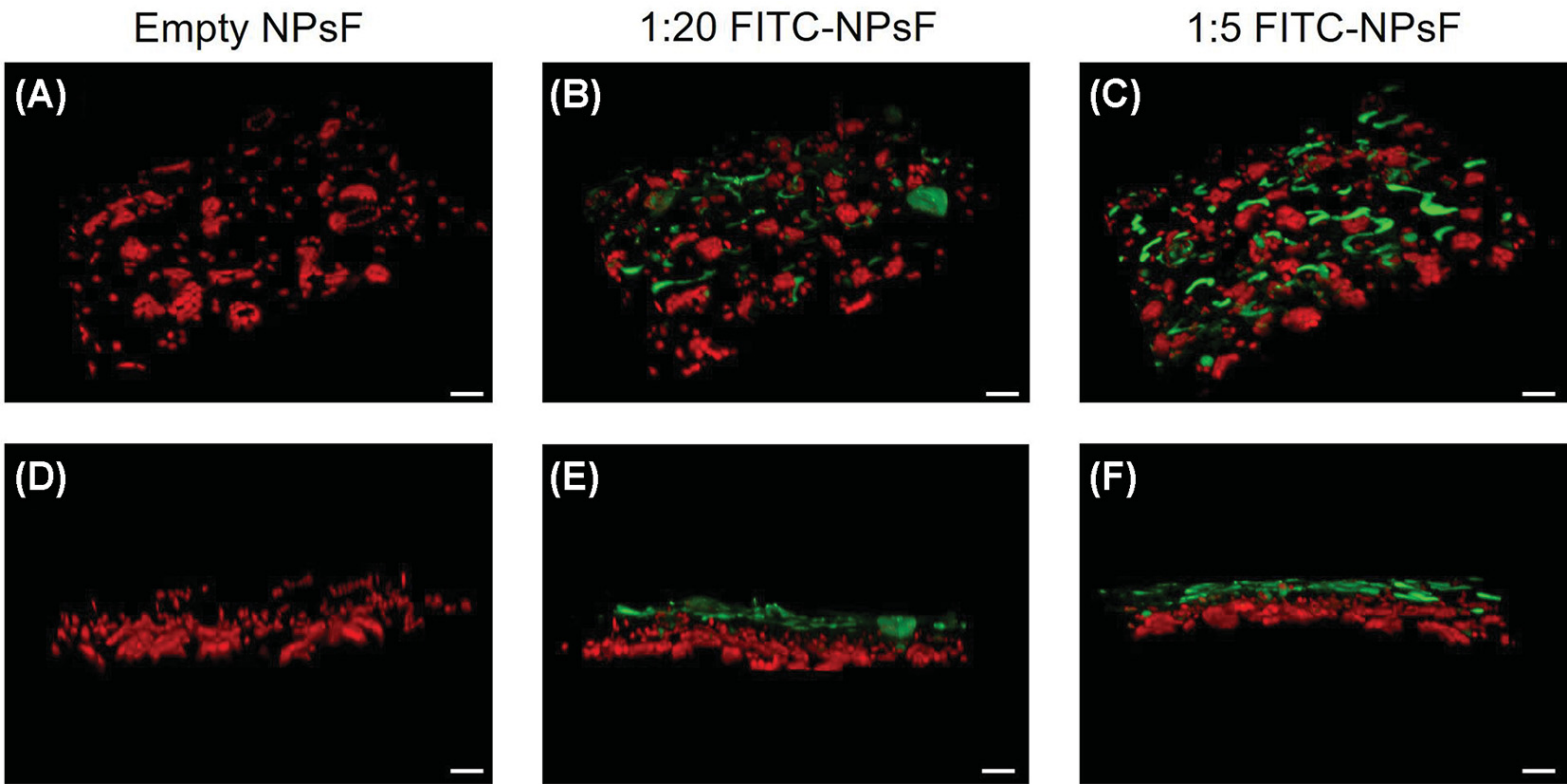
3D reconstructed confocal micrographs showing the distribution of NPsF in 0.1% EIA on *N. benthamiana* leaf abaxial surface Tilted and side views spanning from the leaf epidermis to the mesophyll are reported. Panels (**A,D**), empty NPsF; panels (**B,E**), 1:20 FITC-NPsF; panels (**C,F**), 1:5 FITC-NPsF. Green, FITC; red, chlorophyll autofluorescence; scale bar: 20 μm.

The 3D reconstructions of the *N. benthamiana* leaves shown in Figure 4 confirmed NPsF accumulation and the correlation between the titre of the NPsF suspension used in the treatment and the amount of fluorescence detectable on leaf surface (Figure 4; see also Supplementary Figure S5). Notably, the 1:1 dilution of the nanomaterial exhibited a quite complete covering effect, which probably led to an unspecific incrustation of tegument cell wall (Supplementary Figure S5). In addition, the 3D views highlight the predominantly external localisation of NPsF due to virtually absent fluorescence signal below the epidermis. This is an expected result given the short time interval (max 4 h) between treatment and microscopy analysis, and at the same time is a demonstration of the FITC-retention capacity of the nanomaterial in comparison with free FITC (see Supplementary Figure S6).

**Figure 5:**
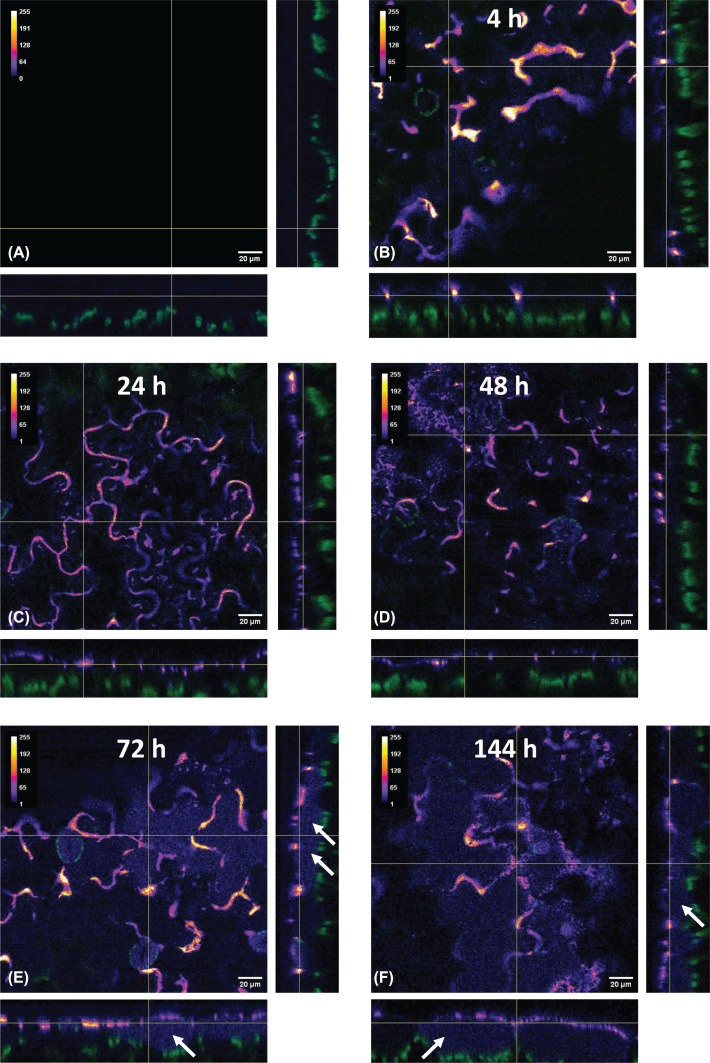
Time course analysis after FITC-NPsF application on *N. benthamiana* leaves Orthogonal views of confocal microscopy z-stacks on leaves treated with FITC-NPs diluted 1:20 in 0.1% EIA. The Fire heatmap lookup table was applied to display FITC fluorescence signal (yellow to purple, highest to lowest intensity); green: chloroplast autofluorescence. Panel (**A**): Control (0.1% EIA only); panels (**B-F**): FITC-NPsF 4 (**B**), 24 (**C**), 48 (**D**), 72 (**E**) and 144 h (**F**) after application. Arrows highlight FITC signal inside epidermal cells; scale bar: 20 μm.

After the choice of the most suitable concentration of FITC-NPsF (dilution 1:20), a further test was carried out to study the fate of nanoparticles that remained in contact with the leaves for up to 6 days. The results, based on the indirect evidence of the fluorescent signal of FITC probe, showed that the distribution of NPsF around the cell borders tended to spread evenly starting from 24 h after application (Figure 5C). Nanoparticles remained largely on the leaf surface until the end of the experiment, but after 72 h a faint fluorescent signal was also detected inside of the epidermal cells, reaching the chloroplast zone in the mesophyll, as observed in Figure 5E,F.

Finally, trials were conducted to evaluate the efficacy of the dsRNAs-NPsF combination.

**Figure 6:**
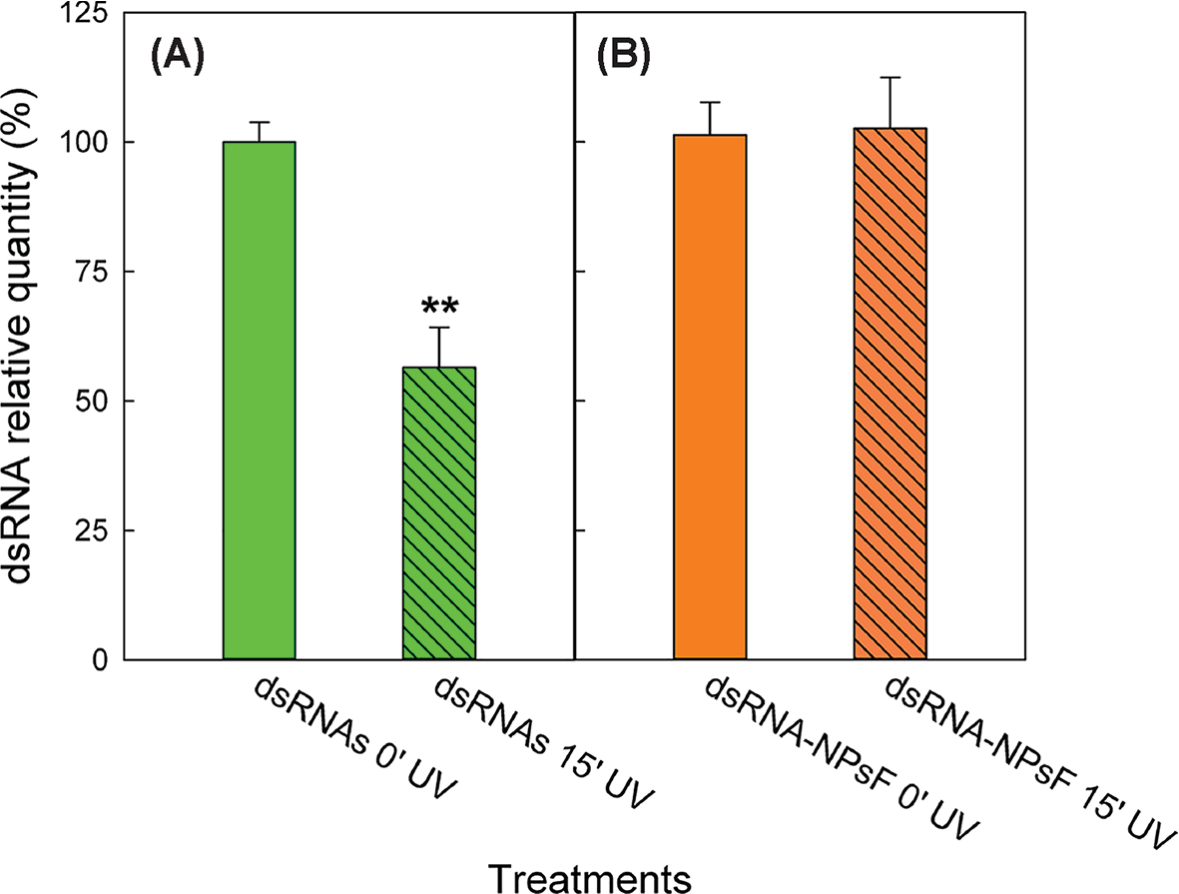
UV disrupting effect Relative *GFP* dsRNA quantity on *N. benthamiana* leaves after 15 min exposition to UV light: comparison between treatment by 1:20 RNA in 0.1% EIA applied as free soluble form (**A**) and functionalised in NPsF (**B**). Data are mean ± SD (*n*=3). The significance of the applied *T-*test is **P-value < 0.01.

Firstly, NPsF were applied to detached leaves from *N. benthamiana* plants to assess capability of chitosan nanomaterial to protect dsRNAs from degradation by environmental agents and to improve their persistence on foliar surface. The first test, consisting in the exposition of NPsF-treated leaves to UV light, demonstrated that functionalisation in NPsF decreased the sensitivity of *GFP* dsRNA to denaturing radiation. In fact, as seen in Figure 6, the free genetic material sprayed on leaves was significantly degraded after 15 min of UV exposure, while that included in NPs showed no significant difference if compared with the relative control at time 0. Moreover, the analysis did not detect the presence of nucleotides on leaves exclusively treated with NPsF, thus confirming the absence of contamination during the application phase (data not shown).

On the contrary, the *in planta* test concerning the permanence of *GFP* dsRNA over time, did not show significant differences between the amount of free and NPsF-bound dsRNA detected after 7 and 15 days from application on *N. benthamiana* leaves, respectively (Supplementary Table S1). Indeed, no significant degradation of free *GFP* dsRNA was observed even if a decreasing trend was identifiable in free-RNA-treated samples, suggesting the need for a more prolonged environmental exposure or to replicate the experiment in an open environment.

**Figure 7:**
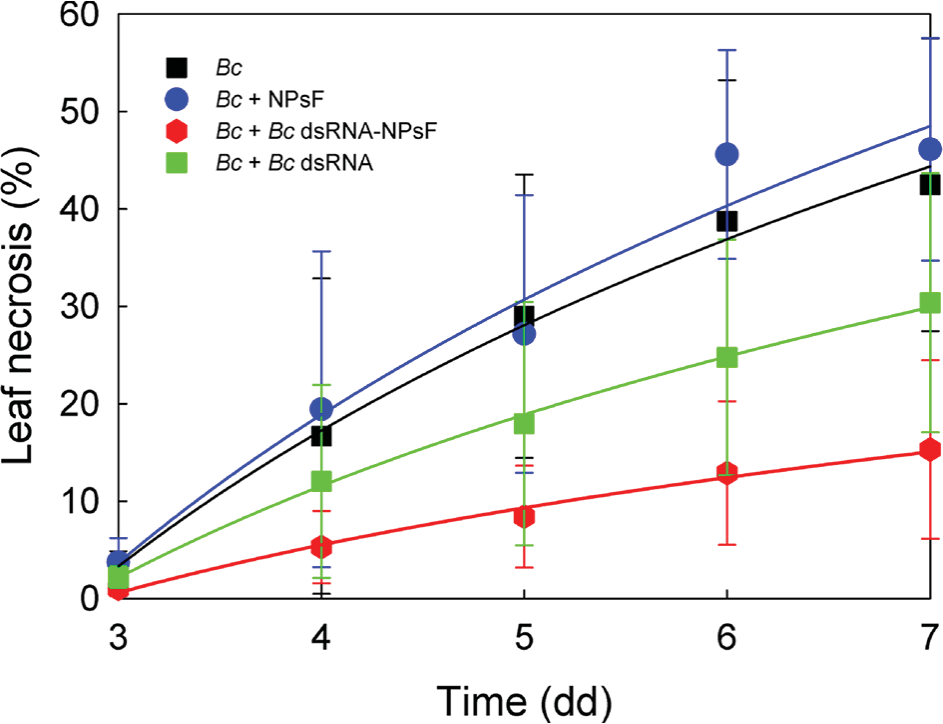
Development of *B. cinerea* necrosis on *N. benthamiana* leaves within 7 days Data are expressed as the average percentage of leaf area invasion ± SD (*n*=12); Bc, Botrytis cinerea.

Finally, a specific nucleotide fragment expressing *B. cinerea* essential genes was employed to assess the degree of inhibition of fungal development on *N. benthamiana* leaves. As shown in Figure 7, naked *Bc* dsRNA was able to reduce mycelium growth in comparison with the control and *Botrytis* diffusion was severely restricted also when the sequence was bound to NPsF, as proven by the significance level of the treatment factor (see Table 1). In addition, the long-lasting and better protective effect exerted by dsRNA NPsF compared with naked dsRNA was confirmed by significance of statistical analysis (Supplementary Table S2). In contrast, the application of empty NPsF had no effect, and necrosis developed similarly to the untreated leaves.

**Table 1:** Growth inhibition of *B. cinerea* mycelium due to the effect of dsRNA interference technique applied on *N. benthamiana* leaves

Response variable	Source of variability	Df	*F*-value	*P*-value (>F)
*B. cinerea* mycelium growth	Time	4	80.63	<2e^−16^ ***
	Treatment	3	41.09	<2e^−16^ ***
	Residuals	229		

Data are expressed as mean ± SD (*n*=12). Significant relationships are in bold. Df = degrees of freedom; *F* = Fisher value; *P* = level of significance. *** indicates a *P* significance level < 0.001.

## Discussion

Raising concerns about environmental variables have boosted the development of strategies able to increase efficacy of pest control by means of smart and low impact approaches.

According to this assumption, the application of dsRNAs as sustainable alternative to synthetic pesticides is a powerful and promising methodology, since it induces the RNAi pathway which is able to silence target genes in invading pathogens.

The rationale of present study is to investigate the characteristics of chitosan-based nanomaterials suitable for dsRNAs encapsulation, contributing to recent advances in chitosan NPs entrapping dsRNA delivery and improving RNAi-based response in plant crop protection [37].

Additionally, such an approach guarantees a protective effect on leaf-applied nucleotide sequences quite sensitive to degradation under field conditions, which limits their protection to less than seven days [38]. The entrapped chitosan-dsRNA NPs could further open the opportunity for future developments involving the ability of nanomaterials to overcome leaf cuticle barrier, by-pass through cell wall and plasma membrane and avoid degrading *in vivo* nucleases [31,37]. Comparably to medical treatments, low-dimension NPs may allow easy and targeted delivery of functionalising molecules also inside plant tissue, possibly giving them free access to cell metabolism and even a long-distance distribution with systemic effects or being more efficiently uptaken by phytopathogens [39].

The most innovative and sustainable solutions require an efficient distribution and delivery of very precious and delicate material such as nucleotide sequences inside the plant, where the presence of a fully hydrophobic leaf tegument and of a cell wall creates specific conditions. Actually, there is a poor knowledge about the mechanisms underlying the different steps of nanomaterial transport in plants, namely: (i) plant surface adhesion and lifetime; (ii) internalisation beyond the tegument barrier; (iii) translocation along both a short and long distance involving passive and active mechanisms and finally (iv) accumulation inside cell after crossing over cell wall and plasmalemma [40]. All these steps are strictly dependent on NP size, concentration and surface charge, which are the main physico-chemical features to be considered in the design of nanomaterial able to provide the best transport efficiency.

The initial approach of the present investigation was then devoted to implement a synthesis protocol for NPs, using ionic gelation method, aiming to minimise NP dimension and to allow nucleotide retention inside NPs at the highest level possible. As reported in literature, in ionic gelation method, an optimal encapsulation of nucleic acids should be usually favoured by their strong cross-linking with TPP inside the chitosan matrix, instead of depending only on electrostatic interactions, thus enhancing their potential retention ability [41,42].

Initially, to obtain small NPs that would be more functional for interaction with the leaf surface, two different synthesis protocols were defined by decreasing the polymer molecular weight of bulk chitosan. This aim has been addressed through two modalities, namely the filtration of the stock solution or its oxidative degradation for 24 h, both defined after the preliminary tests shown in Supplementary Figures S1 and S2. In the first one, filtration lowered the concentration of long chains (average diameter <500 nm), therefore attenuating the effect of intermolecular interactions during synthesis with TPP, that would cause the formation of large NPs [43]. Instead, the degradation process itself decreased the presence of amino groups in favour of carboxyl ones [44] and the size of the chitosan chains [36,44], favouring the synthesis of smaller NPs (<400 nm).

The size of the NPs was studied both before and after functionalisation with RNA: as the NPsFs were considered better in terms of lower size increase after functionalisation (Figure 1) and stronger binding capacity (Figure 2), they were also subjected to a more precise dimensional analysis by TEM (Supplementary Figure S3), which allowed to estimate the actual average particle size of 170 ± 61 nm.

Both NP formulations presented a neutral charge (Figure 1), which represents an optimal feature for enhancing their interaction with hydrophobic leaf epidermis thereby decreasing NP aggregation or loss due to solubilisation by rainwater runoff [45].

The shift in the balance of charges in favour of anions in the chitosan polymers [44], which might have occurred during degradation treatment involved in NPsD synthesis, also negatively affects the ability to bind RNA, being itself a polyanion [43]. This explains the result of gel electrophoresis analysis for complexation of dsRNA with NPs (Figure 2), where NPsD functionalised by RNA showed only a partial complexation, evidenced by a smear similar to that of free nucleotides, while the complex nucleotide-chitosan formation became optimal in the RNA-NPsF sample, where bound dsRNA did not migrate under electrophoresis. Similar results were obtained by Petrônio and collaborators [46], using coacervation synthesis protocol for obtaining small-sized chitosan-dsRNA NPs from high molecular weight bulk chitosan. The more efficient complexation of RNA sequences with NPsF compelled us to choose the filtration method for the synthesis of chitosan NPs in the following plant tests and confocal microscopy analyses.

The electrophoretic analysis provided additional information, since it was possible to demonstrate that the refinement treatments (in particular the sonication) as well as the addition of the surfactant EIA, did not affect the retention capacity of the RNA by the NPs, as confirmed by the absence of non-specific release.

The efficient functionalisation with RNA was further attested by the size modification of the NPs (Figure 1), which represented an additional preferential factor toward NPsF, since they exhibited a smaller apparent hydrodynamic diameter than NPsD. Conversely, the values of the ζ potential were not affected.

In a view to future agronomic application of the NPs thus obtained, the characteristics of a formulation including a surfactant commonly used in agriculture was tested by its distribution on detached leaves of *N. benthamiana*.

With the aim to investigate the deposition and early penetration of the chitosan-based NPs suspension on the abaxial leaf surface, 3D confocal microscopy analysis was performed with small-size FITC-conjugated NPsF suspended in wetting agent and applied by foliar spray. Despite fluorescent-labelled chitosan-NPs have been largely used in medical literature, offering the advantages of tracking the extracellular and intracellular NPs delivery and allowing a stable and high fluorescence signal emission [47], analogous studies in plant systems are still scarce. Microscopic fluorescent NPs imaging showed a dose-dependent adhesion on the abaxial leaf surface and a preferential localisation along the tangential walls of the tegumental cells, in agreement with the evidence of different authors [48–50]. This is more marked in the NP treatments applied at 1:20 and 1:5 dilution ratio as shown in Figure 3 (panels B and E; C and F, respectively) and in Figure 4 (panels B and E; C and F, respectively). On the contrary, the thesis at 1:1 NPsF dilution showed a hedging effect likely due to an excess of large-size aggregates of particles (Supplementary Figure S5), and for this reason it was not taken into consideration for further tests. In fact, the use of high concentrations of nanomaterials sprayed on plant leaf could be counterproductive, since it induces large-size aggregates formation on leaf surface probably explained by the higher probability of particle collision [51], at the expense of NPs’ mono-dispersity (see Supplementary Figure S5), thus ultimately affecting the effective penetration of the NPs inside the tissues and controlling their fate in plant systems.

Furthermore, this would alter the intrinsic properties related to the nanometric size of the materials, such surface charge distribution and high surface:volume ratio, while at the same time would reduce their contact surface available for interaction with the leaf epidermis.

In Supplementary Figure S6, the fluorescence observed upon leaf exposure to free FITC diluted 1:1000 is compared with that obtained after application of the supernatant from low concentration (1:20) of FITC-NPsF, previously washed by centrifugation to get rid of exceeding FITC. The negligible signal in the case of the supernatant, as compared with both the corresponding suspension (see Figure 3) and the free FITC solution, supports the absence of significant dye leakage in the FITC-NP formulation.

Confocal analysis also allowed to observe a partial localisation of FITC fluorescence signal within chloroplast-containing guard cells (Figure 3), suggesting that at least a portion of the NPs could pass through the stomatal rims entering the guard cells. Such fluorescence signal could be due both to intact FITC-NPsF, possibly those belonging to size classes below 20 nm [40], or to dye molecules eventually released from the functionalised NPs, once beyond the tegument barrier. Accordingly, recent evidence reported that foliar uptake of nanoparticles proceeds both via a polar pathway (e.g., trichomes, stomata) and a nonpolar pathway (e.g., cuticle and its pores), depending on their size, charge and form [52], but it is strongly dependent also on the plant-species dependent leaf features and wettability [53,54].

Notably, although this signal in the guard cells was observed also in the case of leaf treatment with free FITC in solution (Supplementary Figure S4a and S4c), in the treatment by NPsF supernatant (Supplementary Figure S4b and S4d) only a negligible fluorescence could be retrieved at stomata level. These results suggest that the minor contribution from leaked dye present in the supernatant (marker of high retention NPs) is unlikely to explain the high signal detected in these cells upon FITC-NPsF application. In addition, the high fluorescence signal mostly associated to the guard cells implies a transport against concentration gradient. Such an activity is explainable by an energy-driven accumulation of FITC-NPsF or free FITC, since a passive accumulation-driven by a concentration gradient is hardly conceivable, given the already cited low fluorescence associated to the supernatant. Instead, it could be inferred that the permeation is limited to guard cells only, since stomata are known as the only epidermal cells able to perform an actual photosynthesis and therefore to synthetise a large amount of ATP [55]. This observation is in accordance with what was recently reported by Landry and colleagues [49], working with different microscopic techniques on DNA-modified gold NPs uptake in *N. benthamiana* leaves. They suggested that, depending on size and shapes, the route for NPs delivery into plant cells is not accomplished by a simple diffusion mechanism, but could be mostly driven by an energy-dependent endocytosis process or even that the particles could accumulate in the cell walls, then releasing their cargo.

It is necessary to specify that this confocal microscopy analysis was carried out over a short period of time (no more than 4 h from treatment). A time-course experiment was subsequently performed to verify the persistence of sprayed NPs on leaf surface during longer time periods.

The images acquired by means of confocal microscope analysis showed the orthogonal view of the outer layers of the leaf cells. Although the indirect approach used, based on the fluorescence of FITC dye used as NPs doping agent, doesn’t allow to discriminate between fluorescence of free released FITC or that of dye still bound to the nanoparticles, the collected images revealed a time-dependent modification of the distribution pattern. In particular, the time-course of images shows that the distribution pattern underwent a time-dependent modification and from an initial localisation preferably along the edge of the cell walls, in agreement with the initial short time analysis, then a more even distribution was observed within 1 or 2 days. Although the experiment was prolonged over several days with light/dark cycles, the fluorescence signal remained detectable until at least day 6, an observation that would suggest a protective effect of presumably still intact NPs against photobleaching of the doping molecule. From day 3 a weak signal was detectable inside the cells of leaf tegument, indicating that at least the free FITC had partially permeated the waterproof layer of the leaf. These results are promising as they show that NPs provide the functionalising molecules with protection against environmental factors (light in particular), which did not result in massive fluorescence loss. The late appearance of the fluorescence inside plant tissues could be explained by a gradual and prolonged release of the doping molecule, occurring concurrently with the slow NPsF degradation. Durable adhesion on the leaf, overcoming the impermeable barrier created by the cuticle and subsequent transport into the cells are currently the weak points for rapid diffusion of RNA interference [54]. These results thus provide an additional strength point for the practical application of this smart strategy.

The promising feature of chitosan nanomaterials to enhance dsRNA stability and delivery on plants with the aim to activate RNAi response was preliminarily verified by treating *N. benthamiana* leaves with dsRNA-functionalised NPsF. Quantification of specific *GFP* dsRNA amount in leaf tissue by means of PCR technique allowed to evaluate the rate of nucleotide degradation both on detached leaves exposed under UV light, mimicking drastic environmental conditions, and on plants in a controlled environment.

In the harsher conditions, under a brief exposure to UV light, *GFP* dsRNA functionalised with NPsFs were actually protected by degradation, while leaf dsRNA content significantly decreased in leaves treated by naked RNA. Accordingly, exogenous spraying of naked dsRNAs for plant virus resistance under field conditions has been already shown to be limited by the high instability and short half-life [38,56] and this poses several controversial discussions on the successful application of exogenous dsRNA for RNAi systems [57], nonetheless several innovative nanosystems for effective RNA delivery have been proposed to overcome this challenge [58].

Unfortunately, the same approach showed no significant difference between the two treatments if applied *in planta* during a 7 to 15 days cultivation period in a growth chamber (Supplementary Table S1). This result confirms on the one hand that the dsRNA molecule on leaf surface has fair stability over time and on the other hand that controlled growing conditions induce a weak environmental effect, which is very different from field conditions.

The obtained data confirmed the initial hypothesis, since dsRNAs appeared to be stable over an acceptable period of time in the perspective of future application as a molecular ‘smart strategy’. This goal was interestingly further amplified since the distribution of functionalised NPsF, although not at a statistically significant level, suggest the presence of a more favourable behaviour in the decay trend of the dsRNA and the possibility of a long-lasting coverage using RNAi technique for protection from pathogen and environmental stresses.

Such a hypothesis has been further demonstrated by the experiment described in Figure 7, where the already known antifungal effect [59] related to RNA interference approach has been further potentiated by NPs functionalization and this strengthen the feasibility of its application in the case of pests and pathogen present on the leaf surface. The observed decrease of the *Botrytis* mycelium diffusion could be almost completely ascribed to a merely protective effect of nanoparticles on nucleotide sequences, without the expected anti-mycotic effect of chitosan. This observation suggests that this smart technology based on RNAi could be even further improved in case the chitosan raw material used for NPs synthesis would exhibit antimicrobial activity by itself. Further experiments should be performed to evaluate different chitosan formulations with greater reactivity to induce an additive inhibitory action, being aware that secondary effects of phytotoxicity must be avoided.

## Conclusions

The present investigation provides a synthesis method for preparing NPs using filtered chitosan as a starting raw material with specific size and surface charge characteristics, which were shown to be useful for topical foliar application, in comparison with similar NPs prepared by oxidative degradation of chitosan. The efficient leaf distribution of FITC-labelled NPs on *N. benthamiana* leaf was analysed by means of 3D fluorescence confocal microscopy, which allowed to evidence that low dilution dose (in particular 1:1 rate) induced aggregation of the nanomaterial and loss of monodispersed distribution. When loaded with dsRNA, NPFs also ensured a stable retention of nucleotides even under an electrophoretic field. Furthermore, foliar topically applied NPFs protected the *GFP* dsRNA sequence from degradation by a brief exposure under UV radiation, although, when distributed in plants grown under climatic room conditions, RNA-functionalised nano-formulation did not significantly differ in RNA protection from naked-RNA treatment. This result could suggest that these molecules exhibited appreciable stability when exposed to mild conditions such as those of a climatic room. dsRNA-chitosan NPs tool opens interesting opportunities for the development of RNAi techniques in plant crops. In particular, this solution could be potentially applied for the pathogen control in high-value crops, in order to comply with new UE regulations, prescribing a lower use of agrochemicals and the decrease in their environmental impact. In the present work we confirmed that *Bc* dsRNA shows an inhibitory action against *B. cinerea*, a pathogen developing outside plant tissues, confirming that NPs functionalisation effectively was advantageous in restricting fungal infection. In addition, we provided indirect evidence that functionalised agent bound to the NPs could permeate at least leaf epidermis, since a fluorescent signal linked to FITC dye has been detected by means of confocal microscope in an in an interval of 3 to 6 days after distribution to plant leaves.

## Experimental procedures

### Plant material

Wild-type plants of *Nicotiana benthamiana* Domin were used for all experiments. A set of six plants has been employed to study the NPsF by fluorescence analysis under the confocal microscope, while to study their long-term fate 10 plants were used. Evaluation of the NPsF ability to enhance the nucleotides lifetime, 18 plants were used in the case of leaf treatment and 27 for the whole plant-application. Finally, the leaves of 48 plants have been employed to study the effect of treatments by dsRNA-NPsF on *B. cinerea*.

The plants were cultivated in standard conditions at 22°C and 14 h of light per day in a growth room. For all purposes the leaves were collected at the 3rd to 5th node, corresponding to the first fully expanded leaves, from plants grown for 60–90 days after germination.

### Synthesis and partial purification of double-stranded RNA (dsRNA) molecules

Synthesis and partial purification of both dsRNAs molecules utilised in the present work were performed as previously detailed [59]. Briefly, specific primers were designed to amplify a 376 bp amplicon from the pCBCT plasmid. Once purified the PCR product was digested with the restriction enzyme PstI and cloned into the linearised L4440 plasmid. The later was introduced into HT115-DE3 *Escherichia coli* cells, which are defective for the RNase III gene, involved in degradation of dsRNAs, and contains T7 RNA polymerase under control of the inducible *Lac* promoter. The modified L4440 plasmid, which contains two convergent T7 promoters, was then exploited to induce the production of dsRNAs in HT115-DE3 cells by adding IPTG at the exponential growth phase. For dsRNAs targeting *B. cinerea* essential genes the plasmid previously produced was exploited [59]. This plasmid contains the sequence of *BcCYP51*, *Bcchs1*, and *BcEF2* obtained by overlapping PCR. Similarly, a single fragment of the GFP coding sequence was independently cloned into the L4440 plasmid in order to obtain dsRNAs with this incorporated sequence.

Extraction of dsRNAs from bacterial cells was achieved through a classic phenol–chloroform extraction followed by isopropanol precipitation and then by DNase I treatment. dsRNA integrity was checked with a 1% agarose gel using a weighted ladder to obtain a semi-quantitative evaluation of the extracted amount.

### Synthesis, functionalisation and refinement of chitosan NPs

Chitosan (Acros Organics, MW: 100,000–300,000 g mol^−1^) was purchased from Thermo Fisher Scientific (Fair Lawn, NJ, U.S.A.); all other reagents were from Sigma-Aldrich Chemical Co. (St. Louis, MO, U.S.A.), if not otherwise specified.

Two types of nanoparticles (NPs) were synthetised, using chitosan stock solution differently prepared. In the first case, it was obtained by dissolving chitosan powder in an aqueous solution of acetic acid (adjusting the pH approximately 3.5), stirring it for more than 12 h and then filtering it with 0.2 μm syringe filters (Nalgene Syringe Filters, SFCA membrane, 25 mm diameter; Thermo Fisher Scientific). For this reason, this kind of NPs are hereinafter referred to as NPsF.

The second stock solution was instead obtained by pre-treating chitosan for 24 h (the treatment duration was determined according to previous assays, see Supplementary Figure S1) with a 6% hydrogen peroxide solution, according to Zhao and Wu method [36], with the aim of degrading it and decreasing its molecular weight. Chitosan was then dried and dissolved in the same solution as above, but without carrying out the final filtration; the thus prepared NPs will be named NPsD.

Both NP types were then obtained by the ionic gelation method [35,60], using TPP (sodium triphosphate pentabasic, MW: 367.86 g mol^−1^) as cross-linker agent and TWEEN®20 as surfactant. The amount of chitosan added for each milliliter of the synthetic mixture was 1.57 mg, and the ratio between low molecular weight chitosan and TPP was 5:2, in agreement with the findings of Yang and co-workers [61]. The reaction was carried out at room temperature in an aqueous solution (final volume: 2.1 ml) by adding the TPP dropwise and then stirring for 1 h.

The synthesis yield was evaluated through the difference between the dry weight of the chitosan used and that of the NP precipitation pellet.

The procedure followed to functionalise the NPs was the same as that for the empty ones by the simple incorporation of the doping solution during stirring, before the TPP addition. In the case of RNAi studies, the sources for the doping solutions were, as mentioned above, total RNAs obtained from a transformed *E. coli* strain able to synthetise: (i) the dsRNA of Green Fluorescent Protein (*GFP* dsRNA) or (ii) a specific fragment expressing *B. cinerea* essential genes (*BcCYP51*, *Bcchs1*, and *BcEF2*. For simplicity it will be called *Bc* dsRNA) [59]. For each milligram of chitosan, 6 μg of *GFP* dsRNA or of *Bc* dsRNA were added. The same concentration was used for all experiments. If necessary, the pH of the synthetic solution was adjusted with acetic acid 2.4 M, buffering it to the usual values of empty NP-preparations (pH 5.0–5.5).

NPsF were also functionalised with another molecule, the fluorescein-5-isothiocyanate (FITC; Merck KGaA, Darmstadt, Germany) to allow visualisation by confocal microscopy. In this case, a 1% stock solution in acetone was prepared and NPsF were loaded with 0.1% FITC taking care to avoid exposure to light as much as possible.

After synthesis, all types of NPs were subjected to a refinement process consisting in two phases of pelleting (centrifugation at 16000 ***g*** for 10 min) and final resuspension in 1 ml of double-distilled water. At last, aiming to improve mono-dispersity, NPs were treated with three sonication sessions of 15 s each, at the power of 50 W (Labsonic 1510; B. Braun, Bender+Hobun, Zurich, Switzerland).

### EIA wetting agent addition

Aiming to formulate NP suspensions suitable for an optimal distribution on the plant canopy, the possibility of using an additive with a tackifier function was also considered. Among the various existing commercial products, ethoxylated isodecyl alcohol wetting agent (EIA, 100 g L^−1^, CIFO Srl, S. Giorgio di Piano, BO, Italy) was chosen since it is already widely used in agricultural practices. After refinement, a sample of NPs was, therefore, resuspended in three aqueous solutions with different EIA concentrations (0.025, 0.05 and 0.1%) and used for the tests of NP characterisation.

### Particle size and ζ potential determination

All the NPs, both empty and functionalised, were analysed to assess their properties. Their size distribution and charge were determined by DLS using the Particle sizer/ζ potential Analyzer PSS Nicomp 380 ZLS (Santa Barbara, California, U.S.A.). The measurement was carried out at room temperature in aqueous solution, using refined NP suspensions as samples (cuvette volume ratio: 100 μl of sample in a total volume of 3 ml).

### TEM analysis and image processing

A NPsF sample was assayed by transmission electron microscopy (TEM) to obtain additional information about their shape and size (see Supplementary Figure S3).

Sample preparation was carried out as follows: the NPsF suspension was mixed with 1% uranyl acetate solution, and 5 μl were placed on the microscope (EM 208 - Philips TEM: Philips Eindhoven, Netherland) copper grid. After a 10 min incubation at room temperature, which was necessary to allow material settlement, the liquid excess was removed with a paper towel and the microscope analysis was performed. The instrument was equipped with a Quemesa (Olympus Soft Imaging Solutions) camera.

Three different fields of the obtained images were then processed by ImageJ software (Java version 1.8.0 322) to measure the diameter of individual particles.

### Gel retardation assay

Electrophoresis (Mini-Sub Cell®GT Agarose Gel Electrophoresis System; Bio-Rad Laboratories, Inc., CA, U.S.A.) was performed to evaluate both the *GFP* dsRNA retention capacity by the two types of NPs and whether the addition of EIA influenced it. NPs obtained from filtration treatment (NPsF) and those made from degraded chitosan (NPsD) were doped with RNA and loaded into the gel wells both before and after the refinement treatment; moreover, their final supernatant was also analysed to verify if the sonication caused the release of the RNA from NPs. Further agarose gel analysis evaluated the same samples resuspended after refinement in three different EIA concentrations mentioned above. In all cases the standard The Ambion® RNA Control 250 (Thermo Fisher Scientific, Fair Lawn, NJ, U.S.A.) was used as a reference for migrating nucleotide polymers, as indicated by the segment corresponding to a molecule of approximately 2000 nucleotides (see Figure 2, images are representative of at least three replicates).

The assay was carried out in agarose gel 1.5% (Agarose Type I, Sigma-Aldrich Chemical Co.) in Tris-borate-EDTA buffer (TBE-1X), using GelGreen® (GelGreen® Nucleic Acid Stain, 10,000X; Merck KGaA, Darmstadt, Germany) to stain the nucleotides, after its dilution 1:10,000 into the molten agarose solution (precast protocol). Each well was loaded with 20 μL of the samples and 5 μL of gel loading buffer (bromophenol blue 0.01% w/v, glycerol 30% v/v). The electrophoresis was performed at 60 V for 100 min; then the gel was exposed to UV light (UV Transilluminator 2000; Bio-Rad Laboratories, Inc., CA, U.S.A.) and a photo was taken by a camera.

### *GFP* dsRNA persistence on leaves

To explore the *GFP* dsRNA persistence [62] following both RNAs application (functionalised or naked) on *N. benthamiana* leaves, three different sprayable preparations were produced, containing NPsF, RNA-NPsF and free RNA diluted 1:20 in 0.1% EIA. The aim was to simulate a real agronomic treatment, and this explains the choice of the suspension concentration (the 1:20 dose is the only one which exhibits a potential practical application, since the amount of dsRNAs represents a limiting factor) and of the EIA dosage (whose value equals to that usually recommended by the manufacturer).

With these preparations, two different experiments were conducted. In the first one, a single-leaf treatment was performed: 500 μl of each suspension were sprayed, by means of a glass dispenser, on the adaxial side of single detached *N. benthamiana* leaves. After complete drying, half of the plant material was subjected to UV light exposure (Lamp type G30T8, power 30 W, radiation peak 253.7 nm, UV yield 13.4 W; Sankyo Denki, Japan) for 15 min, while the other half was kept as a control.

In the second experiment, the distribution was carried out on the entire plant (20 ml for each plant) aiming to evaluate the preservation over time of nucleotides due to their inclusion into the nanomaterial. In this case, samples were collected at 0, 7 and 15 days after the spray application.

In both approaches, for each condition three biological replicates were formed pooling leaves from 9 independent plants (3 leaves from 3 plants × 3 biological replicates).

In all cases, samples were freeze-dried and stored at −80°C until use for the subsequent RT-PCR analysis.

Total RNA was isolated from leaf samples using the Spectrum™ Total RNA Kit (Sigma-Aldrich, St. Louis, MO, U.S.A.) following manufacturer’s instructions and RNA concentration of the extracted samples was quantified at the NanoDrop™ (Thermo Fisher Scientific, Fair Lawn, NJ, U.S.A.). DNase treatment and cDNA synthesis was performed as previously reported using about 300 ng of total RNA [62–64]. The absence of genomic DNA contamination was checked before cDNA synthesis by qPCR using specific primers of *N. benthamiana COX* (For: 5′-CGTCGCATTCCAGATTATCCA-3′, Rev: 5′-CAACTACGGATATATAAGRRCCRRAACTG-3′) [65]. RT-qPCR reactions were carried out in a final volume of 10 μl containing 5 μl of SYBR® Green Master Mix (Bio-Rad Laboratories, Inc., CA, U.S.A.), 5 μM specific primers of *GFP* (For: 5′-GTGACCACCCTGACCTACGG-3′, Rev: 5′-CTCCTGGACGTAGCCTTCGG-3′) and 1:10 of diluted cDNA. Reactions were run in the CFX 96 apparatus (Bio-Rad Laboratories, Inc., CA, U.S.A.) using the following program: 10 min preincubation at 95°C, followed by 40 cycles of 15 s at 95°C, and 30 s at 60°C. Each amplification was followed by melting curve analysis (65–94°C) with a heating rate of 0.5°C every 15 s. All reactions were performed with at least two technical replicates. The relative quantification of *GFP* transcripts was quantified after normalisation over the tissue quantity using *NbCOX* housekeeping gene. Gene expression data were calculated as expression ratio (Relative Quantity) to naked dsRNA application at time 0.

### *B. cinerea* inhibition with *Bc* dsRNA-functionalised NPsF

For *B. cinerea* inhibition test, preparations of naked *Bc* dsRNA, NPsF and *Bc* dsRNA-functionalised NPsF were used, all diluted at 1:20 ratio in 0.1% EIA solution. The EIA-only solution was used as a control. A volume of 500 μl of each suspension was sprayed as described above on the adaxial page of 12 leaves, which, once dried, were placed in petri dishes prepared with water agar (1%, w/v). Each leaf was then inoculated with 10 μl of *B. cinerea* spores (1.28E+06 spores per ml) in 30% glycerol, divided into two drops. *B. cinerea* isolate was obtained and maintained on Potato Dextrose Agar as described by Vuerich et al. [66].

The plates were then placed in a humidity chamber and kept in the plant growth room for one week.

To detect the extent of fungal development, photographs were taken from day 3 to 7. A camera placed on a stand at a fixed distance was used, while the plates were backlit to highlight the necrotic spots in respect to the leaf surface. Images were processed with ImageJ software (Java version 1.8.0 322) *Labkit* plugin [67], in order to measure the fungal symptoms by means of a segmentation process. The results were expressed as a percentage of affected leaf area on total leaf area.

### Confocal microscopy

For the initial confocal microscopy analysis, three different FITC-NPsF concentrations were tested by diluting the sample at 1:1, 1:5 and 1:20 ratio, each in the presence of 0.1% EIA. Three other preparations, diluted with the same amount of surfactant as well, were used as controls: empty NPsF (concentration 1:1), 1:1000 diluted (w/v) free FITC (mimicking the loading concentration used in 1:1 NPsF) and the supernatant from the 1:20 FITC-NPsF suspension. The latter was chosen since it was the dilution applied also for all the treatments concerning NPsF functionalised with dsRNAs. Five hundred microlitres of each preparation have been sprayed, by means of a glass spray dispenser, on the abaxial side of a single *N. benthamiana* leaf immediately after detachment from distinct plants. Leaves were mounted for confocal examination directly after complete drying of the treatments.

Briefly, a disk of approximately 1 cm^2^ area was excised from each leaf, placed on a microscope slide and sealed, covered by a film of double-distilled water, under n. 1 thickness coverslips using coverslip spacers and two-component silicone glue (Twinsil® Picodent, Wipperfürth, Germany).

For the time-course experiment, five leaves were treated as described above using the FITC-NPsF preparation diluted 1:20 in 0.1% EIA, while five others were used as controls (sprayed with 0.1% EIA only). The treated leaves were kept in the plant growth room (14-10 h light–dark cycle) in 1% water agar (1%, w/v) Petri dishes for different periods of time (4, 24, 48, 72 and 144 h) before a leaf disk was excised for slide mounting.

Confocal analysis was carried out on a Leica TCS SP8 confocal microscope (Leica Microsystems, Wetzlar, Germany) equipped with a tunable white light laser source set to 495 nm for FITC excitation and to 633 nm to allow visualisation of chlorophyll autofluorescence. Z-stack images, starting at the abaxial epidermal surface, were collected at 0.424 μm intervals using a Plan-Apochromat 40×/1.10 NA water immersion objective. For 3D reconstructions, the total z-scanning range was between 30 and 40 μm, largely depending on different coplanarity of each specimen, to cover an anatomical portion extending from the leaf surface to the mesophyll. Alternatively, thinner stacks recorded over approximately 10–20 μm through the epidermal layer were processed as maximum intensity projections and overlaid with corresponding single-plane brightfield images to allow visualisation of cell boundaries. Micrographs were contrast-adjusted and, for brightfield images only, subjected to gamma correction to improve visibility (gamma value = 0.5).

For the time-course analysis, orthogonal views were generated from approximately 50 μM-thick z-stacks and displayed using a heatmap lookup table to better reveal low intensity FITC signals inside the leaf tissue. Image processing was performed using Leica Application Suite X (LAS X) 3.5.5 software and *ImageJ Fiji* software (Java version 1.8.0 322) [68].

### Statistical analyses

Inferential statistics were performed by means of the software *Statistica*™ release 11.0 (StatSoft Hamburg, Germany) and *RStudio* (2022.02.0-443 version). ANOVA or *T-*test was employed to analyse data and a least significant difference (LSD) test at *P*<0.05 was utilised to evaluate mean values of different treatments.

## Supplementary Material

Supplementary Figures S1-S6 and Tables S1-S2

## Data Availability

The data that support the findings of this study are available from the corresponding author upon reasonable request.

## Abbreviations

DLS: Dynamic Light Scattering
EIA: ethoxylated isodecyl alcohol
FITC: fluorescein-5-isothiocyanate
*GFP* dsRNA: Green Fluorescent Protein double-stranded RNA
NP: nanoparticle
NPsD: nanoparticles from degradation treatment
NPsF: nanoparticles from filtration treatment
RNAi: RNA interference
TEM: transmission electron microscopy
