# Supplementary material for: Characterisation and functionalisation of chitosan nanoparticles as carriers for double-stranded RNA (dsRNA) molecules towards sustainable crop protection
Source: Biosci Rep. 2023 Nov 10;43(11):BSR20230817. doi: 10.1042/BSR20230817 (PMC10643051; doi:10.1042/BSR20230817)
Supplement: Supplementary Figures S1-S6 and Tables S1-S2 [file BSR-2023-0817_supp.pdf]

SUPPORTING INFORMATION

Characterization and functionalization of chitosan nanoparticles as carriers for double stranded RNA (dsRNA) molecules towards sustainable crop protection

Dora Scarpin[1], Luca Nerva[2], Walter Chitarra[2], Loredana Moffa[2], Francesca D'Este[3], Marco Vuerich[1], Antonio Filippi[3], Enrico Braidot[1], Elisa Petrussa[1]

[1] Department of Agriculture, Food, Environment and Animal Sciences (DI4A), University of Udine, Via delleScienze 206, 33100 Udine, Italy.

[2] Research Centre for Viticulture and Enology, Council for Agricultural Research and Economics (CREA-VE), Via XXVIII Aprile 26, 31015 Conegliano (TV), Italy.

[3] Department of Medicine (DAME), University of Udine, P.le Kolbe 4, 33100 Udine, Italy.

Corresponding author: Tel.: +39 0432558792
E-mail address: enrico.braidot@uniud.it

Table S1. Relative quantity (RQ) of *GFP*-dsRNA on leaves detached from treated plants.

Values are weighted to the quantity of *GFP*-dsRNA at day 0 (RQ=1). Data are expressed as mean ± SE (n=3).

Days after treatment	Treatment	*GFP*-dsRNARQ
7	EmptyNPsF	0.02 ± 0.005
7	Naked RNA	0.93 ± 0.203
7	NPsF-RNA	0.96 ± 0.178
15	EmptyNPsF	0.02 ± 0.004
15	NakedRNA	0.79 ± 0.140
15	NPsF-RNA	0.94 ± 0.205

Table S2. T test between different treatmentson *N.benthamiana* leaves infected by *B. cinerea*.

Data are expressed as mean ± SD (n=12) and were compared bycoupled double tail T test.

Treatment comparison		T test significance
Bc	*Bc* + NPsF	0.951
Bc	*Bc* + *Bc*dsRNA-NPsF	8.027E-11
Bc	*Bc* + *Bc*dsRNA	8.995E-06
Bc +NPsF	*Bc* + *Bc*dsRNA-NPsF	1.867E-10
Bc + NPsF	*Bc* + *Bc*dsRNA	0.001
Bc +*Bc*dsRNA-NPsF	*Bc* + *Bc*dsRNA	2.072E-07

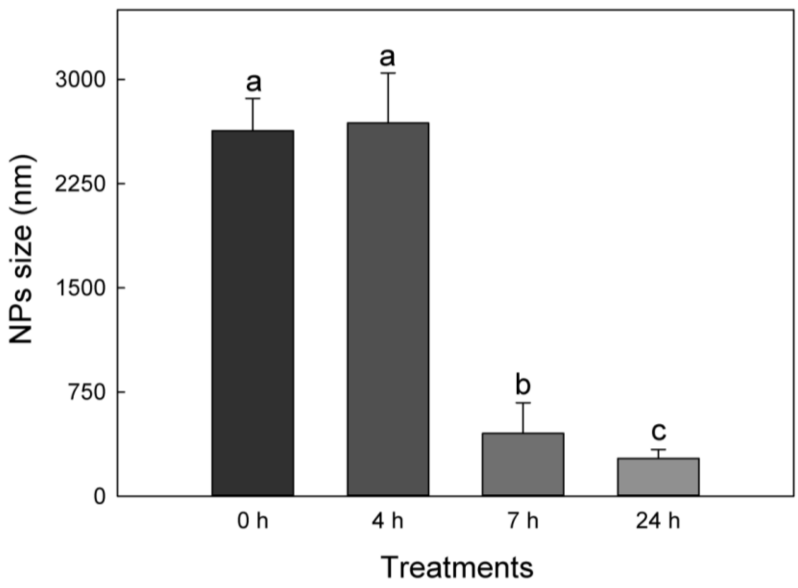

Figure S1. Effect of time duration of the chitosan degradative treatment on NP hydrodynamic diameter.

Data are expressed as mean ± SD (n = 6). Values with different letters are significantly different at P > 0.05 by *post-hoc* LSD test.

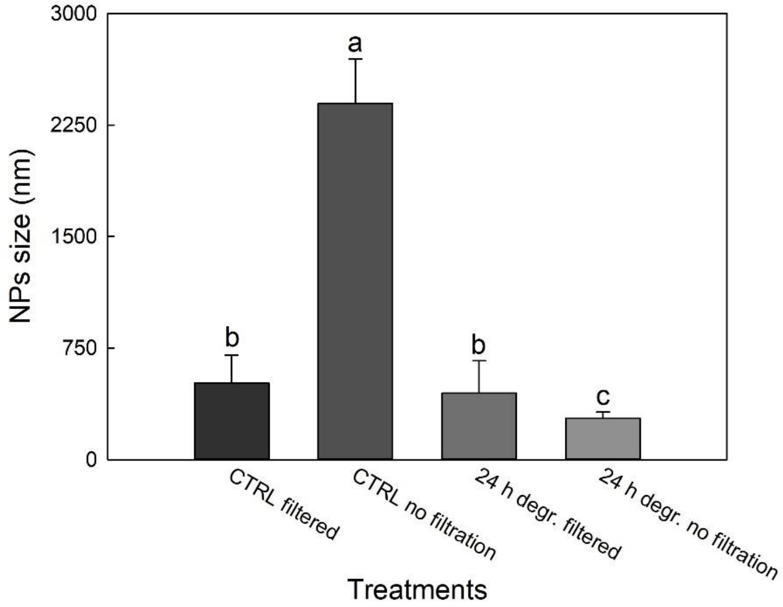

Figure S2. Effects in the size of NPs given by filtration and 24h-H_2O_2 degradation treatments on chitosan stock solution.

Data are expressed as mean ± SD (n = 6). Values with different letters are significantly different at P > 0.05 by *post-hoc* LSD test.

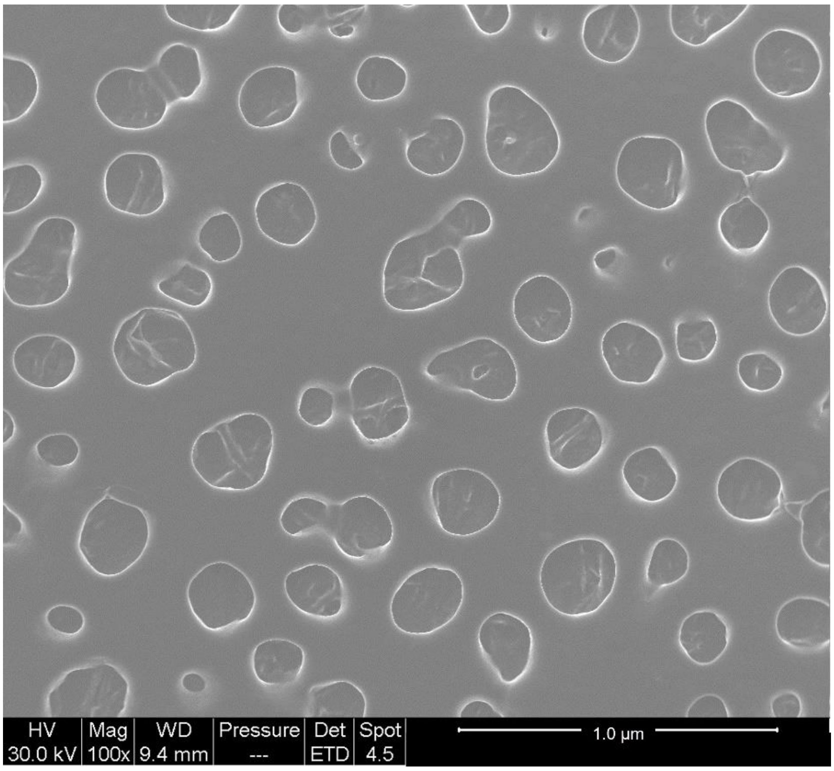

Figure S3. Image of NPsF acquired by transmission electron microscopy (TEM).

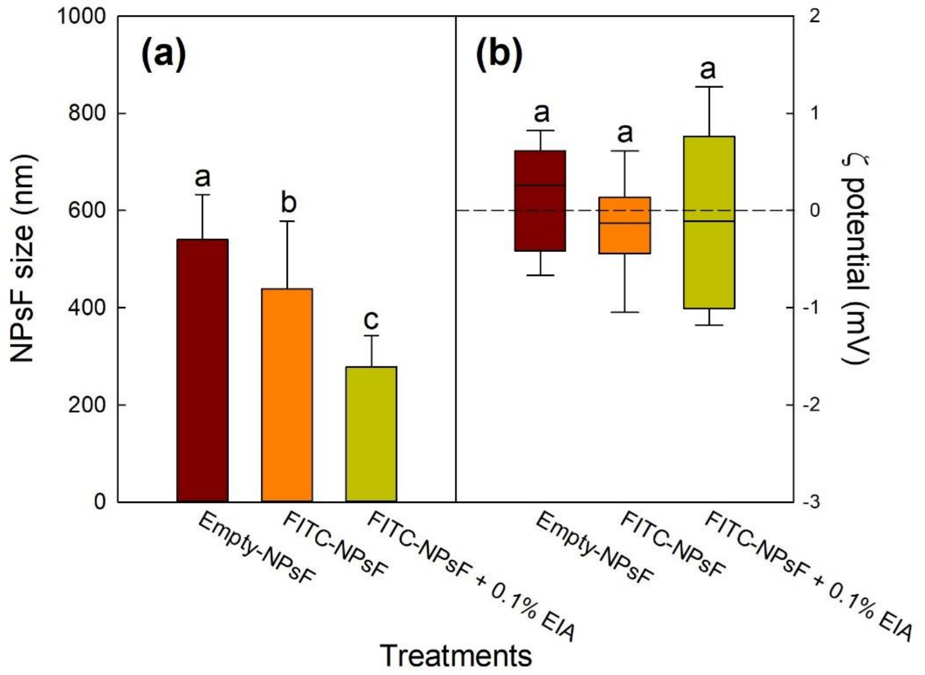

Figure S4. Determination of size (a) and ζ potential (b) of NPsF.

Treatments were: NPsF as such, FITC-functionalized NPsF and FITC-functionalized NPsF suspended in 0.1% EIA solution. Data ($n = 9$) are expressed: (a) as mean ± SD; (b) as boxplot whose whiskers correspond to the data range between minimum and maximum value, excluding outliers. Values with different letters are significantly different at $P > 0.05$ by *post-hoc* LSD test.

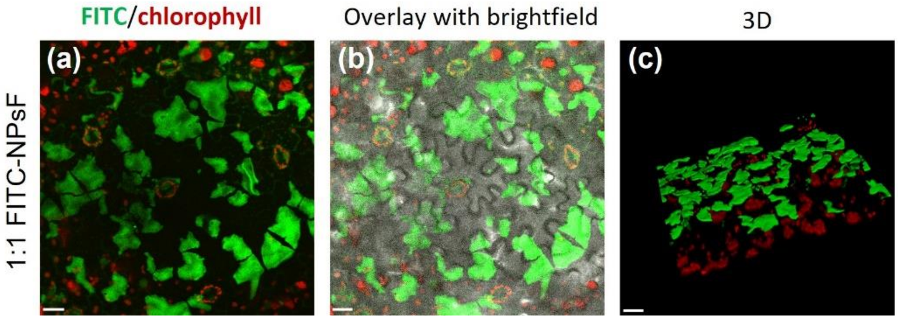

Figure S5. Confocal microscopy analysis of *N. benthamiana*leaves.

Abaxial side of leaf tegument was sprayed with FITC-NPsF diluted 1:1 in 0.1% EIA. (a): Maximum intensity projection (epidermal layer); (b): projection overlay with corresponding brightfield image; (c): 3D rendering (epidermis to mesophyll; different field). Green, FITC; red, chlorophyll autofluorescence. Scalebar 20 µm.

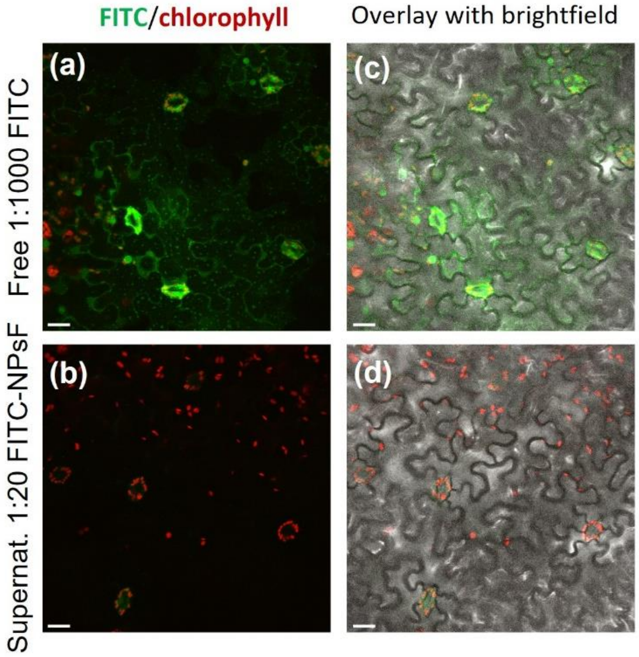

Figure S6. Maximum intensity projection confocal imagesof *N. benthamiana* leaves.

N. benthamiana abaxial side of leaf teguments was sprayed with (a, c) free FITC solution (1:1000) or with (b, d) the supernatant of FITC-NPsF diluted 1:20 in 0.1% EIA (green, FITC; red, chlorophyll autofluorescence). Right column: projections are overlaid with corresponding brightfield images. Scalebar 20 µm.
